# Managing tooth wear with respect to quality of life: an evidence-based decision on when to intervene

**DOI:** 10.1038/s41415-023-5620-4

**Published:** 2023-03-24

**Authors:** Shamir B. Mehta, Bas A. C. Loomans, Roos M. F. van Sambeek, Tatiana Pereira-Cenci, Saoirse O’Toole

**Affiliations:** 41415133911001 Department of Dentistry, Radboud University Medical Centre, Radboud Institute for Health Sciences, Nijmegen, The Netherlands; Faculty of Dentistry, Oral and Craniofacial Sciences, King´s College London, Guy´s Campus, London, UK; College of Medicine and Dentistry, Birmingham Campus, Ulster University, UK; 41415133911002 Department of Dentistry, Radboud University Medical Centre, Radboud Institute for Health Sciences, Nijmegen, The Netherlands; 41415133911003 Faculty of Dentistry, Oral and Craniofacial Sciences, King´s College London, Guy´s Campus, London, UK

## Abstract

Patients with more severe forms of tooth wear may require restorative rehabilitation. The decision to commence treatment must be taken carefully and there are a multitude of factors to consider. Alongside the clinical signs and symptoms typically associated with tooth wear, there is also the need to assess the impact of the condition on the patient's oral health-related quality of life. As part of the discussions relating to the attainment of informed consent for the restoration of the worn dentition, not only is it relevant to appropriately appraise the risks, benefits, costs, reasonable alternatives and likely prognosis of the proposed treatments, but to also elaborate on the expected impact of the intervention on the patient's oral health-related quality of life. The aim of this article is to review the evidence relating to the impact of the quality of life with the management of tooth wear, with the introduction of the concept of an evidence-based approach to decision-making when planning care.

## Key points

- Reviews the evidence relating to the effects of tooth wear on oral health-related quality of life and the available information about the impact of restorative intervention on oral health-related quality of life.
- Gives an insight into how the time and costs associated with tooth wear rehabilitation may be barriers.
- Highlights the need for new guidelines for restorative intervention for patients with tooth wear, with an emphasis on a shared decision-making approach.

## Introduction

Tooth wear is usually the result of the effect of multiple aetiological factors and a plethora of mechanical and chemical causative factors have been reported.^1^^,^^2^ With a mean estimated global prevalence of erosive tooth wear of up to 45% in permanent teeth,^3^ signs of tooth wear with varying levels of severity are likely to be frequently encountered in general dental practice. Patients with severe tooth wear may report symptoms such as impaired oro-facial aesthetics, challenges with effective phonetics and mastication, and/or pain and discomfort.^4^ Aesthetic concerns have been described as the most common reasons for patients with tooth wear to be referred to secondary care settings and concerns with appearance and function may often motivate patients to seek professional help.^5^^,^^6^

Alongside physical wellbeing and the absence of disease or infirmity, there is also the need to consider psychological and social wellbeing.^7^ Conditions involving the oral cavity may adversely impact on occupational prospects, social acceptability and inter-personal relationships, as well as levels of self-confidence and self-esteem.^8^ Given the biological and functional roles attributed to the oro-facial structures, the presence of conditions with clear physical manifestations, such as tooth wear, may also culminate in negative emotional or social consequences. The term 'oral health-related quality of life' (OHRQoL) is used to refer to the impact of oral health or disease on an individual's daily functioning, wellbeing, and overall quality of life. However, as OHRQoL may also be influenced by subjective factors, such as personal feelings, perspectives, personality traits, or opinions, patients with similar clinical levels of tooth wear may report significantly different effects on their OHRQoL.^9^ When planning dental care, it would seem prudent to consider the patient's self-perception of their oral health, together with any clinical findings.^10^

Many patients with tooth wear may be effectively managed by a targeted preventive approach with appropriate counselling and monitoring, emphasising the importance of early diagnosis, risk assessment and appropriate care planning.^4^^,^^11^ In the presence of pathological and/ or severe tooth wear, restorative intervention may, however, be required. A range of materials and methods for the rehabilitation of the worn dentition have been described in the contemporary literature.^12^ Some data relating to the clinical performance of restorations for the treatment of tooth wear are also available.^13^^,^^14^^,^^15^^,^^16^^,^^17^ However, determining when it may be most appropriate to initiate restorative intervention, may be challenging. The latter should consider several other factors beyond the clinical presentation to include the likely treatment time and financial costs of the proposed treatment, as well as the impact of the tooth wear on the patient's OHRQoL. The attainment of valid informed consent for restorative rehabilitation of the worn dentition should also include an appropriate appraisal of the merits, risks, likely prognosis of the proposed treatment and maintenance needs, as well as the potential impact of the intervention on the patient's OHRQoL. Where possible, the temptation to thrust a patient into unnecessary restorative treatment must be resisted.^4^^,^^11^

The aim of this paper is to review the evidence relating to the effects of tooth wear on OHRQoL, as well as the available information concerning the impact of restorative intervention on the OHRQoL and elaborate on the importance of the patient-led decision with treatment planning for the worn dentition. Such information may help provide an evidence-based approach to decision-making relating to the initiation of restorative rehabilitation of the worn dentition.

## The effect of tooth wear on OHRQoL - a review of the evidence base

Several questionnaire-based tools have been developed to measure the quality of life related to oral health. Among these are the Oral Health Impact Profile (OHIP), the Oral Impacts on Daily Performance (OIDP) and the Dental Impacts on Daily Living (DIDL).^18^ Many of these tools are based on Locker's interpretation of the World Health Organisation's model of health, with five consequences of oral disease: impairment; functional limitation; pain/discomfort; disability; and handicap.^19^

Using the DIDL, among a cohort of 76 tooth wear patients and an analogous number of control subjects, Al-Omiri *et al.*,^20^ observed tooth wear patients to be nine times more likely to report dissatisfaction with their teeth in general compared to the control subjects. Significantly higher levels of dissatisfaction with all five domains of the DIDL were also observed among the tooth wear group. Of note, the levels of dissatisfaction expressed were independent of the tooth wear severity or any personal factors.

**Fig. 1:**
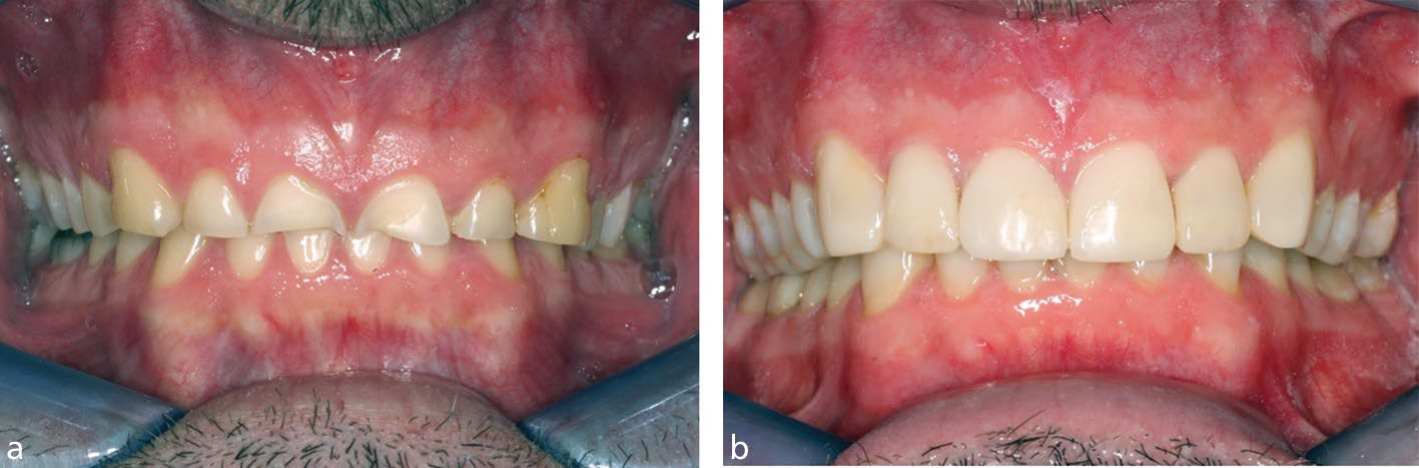
a, b) Restorative intervention may improve the quality of life with severe tooth wear in the anterior region. Pictures from the Radboud Tooth Wear Project, Nijmegen, The Netherlands

The OHIP-49, comprising 49 statements (or focused versions of the OHIP adapted for tooth wear) has also been used in several investigations to evaluate the impact of tooth wear on OHRQoL.^10^^,^^21^^,^^22^^,^^23^ Three of these investigations have recorded a negative impact of tooth wear on OHRLoQ. In 2020, Mehta *et al.*,^10^ reported higher levels of tooth wear (assessed using the Basic Erosive Tooth Wear Examination) to be significantly associated with a deteriorating OHRQoL among a sample of 319 new dentate adult patients attending a general dental practitioner in either Malta, the UK, or Australia. In this study, a focused version of the OHIP - the OHIP-26 - was used. Also applying a shorter and focused version of the OHIP-49 - the OHIP-14 - a previous study using data from the UK 2009 *Adult dental health survey* (5,654 participants), similarly observed a negative impact between the presence of severe tooth wear affecting anterior teeth and the psychological impact of the condition (domains of psychological discomfort and psychological disability).^21^ The questions included in the psychological domains, thus, feeling self-conscious or tense and difficulty relaxing and embarrassment, may have been related to the poor appearance of the aesthetic zone, which is sometimes associated with the presence of severely worn anterior teeth. A restorative treatment may therefore have a positive effect on the quality of life (Fig. 1).

The original English version of the OHIP-49 has also been translated and validated into other languages. Using a Dutch version of the OHIP-49 - OHIP-NL - a negative impact from tooth wear was also reported on patients' quality of life.^22^ Furthermore, the impact of tooth wear was not significantly different to the impact of edentulousness; however, the impact of painful temporomandibular disorders was higher. In contrast, an investigation involving a convenience sample of university students using the OHIP-49 reported the absence of any significant differences between the presence of tooth wear (irrespective of the severity) and the overall OHIP score. However, higher domain scores for functional limitation were observed among participants with severe tooth wear.^23^ University students are perhaps less likely to have longer-term experience of the effects of tooth wear compared to a general population. A more recent investigation using the OIDP noted the presence of a complex relationship between the quality of life, personality and general psychological wellbeing among subjects with tooth wear. In the latter study, reduced levels of general psychological wellbeing and increased levels of neuroticism had independent effects on the quality of life, independent of tooth wear severity.^9^ This makes sense when considering that similar levels of tooth wear will impact patients very differently.

While it would be imperative to consider the clinical findings and the impact on OHRQoL when deciding if and when to commence restorative rehabilitation of the worn dentition (noting that the OHRQoL may not consistently be related to the severity of the presenting tooth wear), it is likely that the decision-making process will require an understanding of broader factors (listed above).^9^ Some of these factors may fall beyond the scope of a dental professional. However, for some patients, where the rate of tooth wear is excessive for age and a clear diagnosis has been made, earlier intervention (especially using minimally invasive techniques) may help to not only yield significant improvements in their OHRQoL, but also help to protect the residual tissues from further wear. This may also optimise the restorative outcome, where the further loss of healthy tissue may otherwise compromise the longevity of the restorative intervention by rendering bonding less predictable,^24^ and perhaps, ultimately, necessitate the prescription of more costly and invasive treatment protocols.

When using tools such as the OHIP (or focused versions), it is also important to note that this may not always give exclusivity for a particular dental condition; an example may include the presence of a discoloured anterior tooth in a patient with signs of severe tooth wear reporting aesthetically related concerns.

## The impact of restorative intervention on OHRQoL

As discussed above, tooth wear may have a substantial negative impact on the patient's quality of life. The effect may be marked, for instance, among patients with tooth wear related to an eating disorder or in the case of an adolescent patients with addiction to erosive beverages. For a proportion of these patients (especially where the pattern of wear may be limited to a more localised presentation), treatment may be successfully provided in a time- and a cost-effective manner, using minimally invasive techniques. Together with the likely survival and success of the proposed restorative treatment intervention, it is also important to consider the benefits a patient with tooth wear may expect to enjoy with improvements in their OHRQoL post treatment.

While the available information is limited (and longer-term information is currently unavailable), significant improvements in OHRQoL and in oro-facial appearance were observed among a sample of patients referred to a Dutch dental university, one year after the completion of full rehabilitation using composite resin restorations. Parameters were assessed using two questionnaires: the OHIP-NL and the Orofacial Esthetic Scale.^25^^,^^26^ The reported outcomes in this study may have been related to the improvements in self-confidence, which may have been previously compromised due to impairment of the patient's aesthetic zone, as well as a reduction in symptoms of pain and sensitivity that may be expected following the application of restorative material across worn tooth surfaces.^26^ The presence of symptoms of pain has been previously reported to be associated with higher OHIP scores, with higher OHIP scores usually indicative of an undesirable effect.^27^ A separate investigation noted significant improvements in the self-perception in the quality of speech function.^28^ In this study, the Dutch Speech Handicap Index was used to evaluate changes following full-mouth occlusal rehabilitation for tooth wear using direct and indirect resin composite, where a mean increase in the vertical dimension of occlusion (VDO) of 2.7 ± 0.73 mm and a mean increase in the length of the maxillary central incisor teeth of 2.6 ± 1.2 mm were provided.

Applying a focused version of OHIP-49 questionnaire, Kalaykova *et al.* in 2019^29^ noted self-reported (subjective) improvements in the ability to eat and chew following direct, full-mouth, composite resin restorations for the treatment of generalised tooth wear. Treatment involved a planned increase in the VDO with marked changes to the occlusal anatomy. However, when using a comminution test (objective) to evaluate masticatory performance, no significant differences were seen between the baseline scores and one-month post treatment with the level of breakdown of a food bolus. This highlights the importance of the individual's psychological interpretation about the impact of tooth wear on their life.

In the above investigations, treatment provision was undertaken by experienced operators. However, information relating to the impact on the post-treatment OHRQoL of tooth wear patients, who may have received restorative care where the performance of the restorations may have been less successful, is unknown. Furthermore, the precise relationship between the severity of the presenting wear and the level of change in the OHRQoL remains to be established; this is an area of further research. Hopefully, in the future, longer-term data, as well as data relating to the impact of other forms of restorative treatments for the rehabilitation of tooth wear (such as the prescription of indirect, fixed restorations, removable dentures and treatment plans involving a combination of differing types of restoration for a patient), will also become available and aid the overall decision-making processes.

## The cost of tooth wear rehabilitation

For many patients, the time required to complete the proposed treatment plan and the cost of intervention may be important barriers for care. Pre-treatment discussions should also include a clear appraisal of the maintenance requirements. Treatment of the worn dentition may be highly time-consuming and technically challenging. Loomans *et al.,* in 2018,^30^ documented the need for five to seven (up to three hour) treatment sessions, including intake and registrations, to enable the execution of full mouth rehabilitation with direct resin composite application. A similar number of appointments were also needed for full mouth rehabilitation using indirect 3D computer-aided design/computer-aided manufacturing nano ceramic restorations.^31^

A service evaluation by O'Toole *et al.* in 2018^32^ reported an average treatment time of 20.8 months for the prosthodontic rehabilitation of severe erosive tooth wear within an NHS hospital setting (range: 8-44 months), with the need for 8-48 clinical visits (mean: 24.3 visits). Treatment sessions were generally 1.5 hours long. This study also reported the total cost for the completion of the treatment plan (excluding staff, materials and laboratory overheads) to range from £675 to £4,807 (mean cost: £2,371) and estimated the cost of providing similar treatments under private arrangements by specialist practitioners based in London, UK, to range from £4,737 to £31,224 per patient (mean cost per patient: £13,353). While the costs and treatment scheduling may vary between differing countries, with factors such as the arrangements under which the care is funded having an impact, for many patients, such costs and time constraints will undoubtedly be prohibitive. Furthermore, access to state-funded facilities may also be inconsistent or unavailable, with the risk of increasing the oral health inequality between individuals who may and may not be able to afford the economic cost of care. It would also be important to consider the cost implications of delaying care, especially if this may impact on the prognosis of the intervention and require further and frequent contingency planning.

Currently, there is no information available about the impact on a patient's OHRQoL where treatment for tooth wear may not be readily accessible.

## Conclusions

Restoration of the worn dentition is by no means consistently straightforward. The decision to intervene may be initially clinically led, but the psychological profile of the patient may determine success. For patients where there may be the need to restore a previously rehabilitated worn dentition with the need to prescribe concomitant occlusal changes, deciding the timing of intervention may be less cumbersome. However, under circumstances where the patient is yet to embark on the restorative pathway, the pre-treatment assessment must take into consideration the impact of the patient's tooth wear on their daily quality of life. Pre-treatment discussions should also ensure the patient is appropriately informed about the likely prognosis, the estimated cost and the expected treatment time and the likely maintenance levels of their treatment options, to ensure they have the necessary information to make an informed decision about their healthcare needs. Together with expected clinical outcomes, preliminary discussions should also encompass the possible benefits with any realistic improvements that may be expected post treatment.

The need for new guidelines for restorative intervention for patients with tooth wear, with an emphasis on making shared decisions with the patients supported by appropriate clinical assessment and appraisal, is indicated. This should take into consideration not just the OHRQoL factors, but also the impact of psychological wellbeing improvements with better appearance.

Ultimately, given that tooth wear progression may be effectively prevented, the need for early diagnosis and risk assessment cannot be overemphasised; however, based on the available evidence, there is clear scope for improvement with this.^33^ In the absence of demand for treatment, commencing restorative intervention should be delayed, with the implementation of appropriate counselling and monitoring, using appropriate tools. However, exceptions may apply to this approach, where there may be substantial levels of tooth tissue loss for age which may have an impact on the patient's oral health and quality of life, where the use of additive minimally invasive techniques may offer a conservative, time- and cost-effective approach.
